# New Insights into the Geographic Distribution of *Mycobacterium leprae* SNP Genotypes Determined for Isolates from Leprosy Cases Diagnosed in Metropolitan France and French Territories

**DOI:** 10.1371/journal.pntd.0004141

**Published:** 2015-10-06

**Authors:** Florence Reibel, Aurélie Chauffour, Florence Brossier, Vincent Jarlier, Emmanuelle Cambau, Alexandra Aubry

**Affiliations:** 1 Sorbonne Universités, UPMC Univ Paris 06; INSERM U1135, Immunity and Infectious diseases Center (Cimi-Paris), team 13, Paris, France; National Reference Center for Mycobacteria, Laboratory of bacteriology, Pitié-Salpêtrière Hospital, APHP, Paris, France; 2 University Paris Diderot, IAME, UMR 1137, Sorbonne Paris Cité, Paris, France; 3 APHP, Lariboisière-St Louis–Fernand Widal Hospital, Laboratory of Bacteriology-Virology, Paris, France; 4 INSERM, IAME, UMR 1137, Paris, France; Fondation Raoul Follereau, FRANCE

## Abstract

**Background:**

Between 20 and 30 bacteriologically confirmed cases of leprosy are diagnosed each year at the French National Reference Center for mycobacteria. Patients are mainly immigrants from various endemic countries or living in French overseas territories. We aimed at expanding data regarding the geographical distribution of the SNP genotypes of the *M*. *leprae* isolates from these patients.

**Methodology/Principal findings:**

Skin biopsies were obtained from 71 leprosy patients diagnosed between January 2009 and December 2013. Data regarding age, sex and place of birth and residence were also collected. Diagnosis of leprosy was confirmed by microscopic detection of acid-fast bacilli and/or amplification by PCR of the *M*. *leprae*-specific RLEP region. Single nucleotide polymorphisms (SNP), present in the *M*. *leprae* genome at positions 14 676, 1 642 875 and 2 935 685, were determined with an efficiency of 94% (67/71). Almost all patients were from countries other than France where leprosy is still prevalent (n = 31) or from French overseas territories (n = 36) where leprosy is not totally eradicated, while only a minority (n = 4) was born in metropolitan France but have lived in other countries. SNP type 1 was predominant (n = 33), followed by type 3 (n = 17), type 4 (n = 11) and type 2 (n = 6). SNP types were concordant with those previously reported as prevalent in the patients’ countries of birth. SNP types found in patients born in countries other than France (Comoros, Haiti, Benin, Congo, Sri Lanka) and French overseas territories (French Polynesia, Mayotte and La Réunion) not covered by previous work correlated well with geographical location and history of human settlements.

**Conclusions/Significance:**

The phylogenic analysis of *M*. *leprae* strains isolated in France strongly suggests that French leprosy cases are caused by SNP types that are (a) concordant with the geographic origin or residence of the patients (non-French countries, French overseas territories, metropolitan France) or (b) more likely random in regions where diverse migration flows occurred.

## Author Summary

Leprosy is an old disease that is nearly eradicated from the European continent but not worldwide. The infectious agent, *Mycobacterium leprae*, has a highly conserved genome, and this property has been used to elucidate the route of its dissemination all over the world. At the French National Reference Center for mycobacteria, 20 to 30 leprosy cases are diagnosed every year, mainly in immigrants from endemic countries or in people living in French overseas territories. A phylogenetic analysis was conducted to investigate the relationship between *M*. *leprae* genotypes and the geographical origin of the patients.

## Introduction

Leprosy or Hansen’s disease is a chronic infectious disease caused by *Mycobacterium leprae* that the World Health Organization called to eliminate (elimination being defined as the reduction of disease prevalence to <1 case per 10 000 population) in the ‘90s [1]. The elimination of leprosy had been achieved at a global level in 2002. However, the number of new cases reported worldwide remained stable around 200 000 since 2005, and in 2013, 44 countries still reported more than 100 cases annually [2].

One of the strategies proposed to reduce the disease burden is to detect leprosy cases as early as possible. In developing countries, the diagnosis remains exclusively based on clinical evidence (e.g. pale or reddish patches on the skin and loss or decrease in sensation in the skin patches). The disease can be classified clinically, on the basis of the number of skin lesions, as paucibacillary (1 to 5 lesions) or multibacillary (more than 5 lesions) [3]. In developed countries, bacteriological examination, histopathological or molecular methods such as PCR applied on slit-skin specimens can support the diagnosis [4] whereas these methods are more difficult to implement in developing countries. Autochthonous leprosy disappeared from all Northern and most Southern European countries. However, 20 to 30 leprosy cases are diagnosed every year at the French National Reference Center (NRC) for mycobacteria, mainly in immigrants from endemic countries or in people living in French overseas territories where leprosy is not eradicated [5].

Genotyping methods have been described to study the global and geographical distribution of distinct clones of *M*. *leprae*. Since the *M*. *leprae* genome is exceptionally well conserved and the bacillus is highly clonal, a correlation has been observed between the geographical origin of leprosy patients and SNP profiles [6]. We aimed at determining the SNP genotype of the *M*. *leprae* strains isolated in France. For this purpose, we applied the method described by Monot *et al*. [6], as well as derived methods, for genotyping of the *M*. *leprae* isolates detected in France during the last 5 years.

## Materials and Methods

### *M*. *leprae* isolates

Biopsies containing *M*. *leprae* from 71 distinct patients received at the National Reference Center for mycobacteria between January 2009 and December 2013 were included in the study. These biopsies were sent from medical centers located in metropolitan France (n = 25), but also in French overseas territories (New Caledonia (n = 20), Mayotte (n = 19), French West Indies (Guadeloupe and Martinique) (n = 3) and French Polynesia (n = 4)). The sex ratio (male/female) was 2.4 (50/21). The average age was 40 (range, 14–86) years. In all, 81 biopsies were examined that were comprised of one biopsy from 63 patients, three biopsies from two patients and two biopsies from six patients.

The biopsies were stored at +4°C until DNA extraction which was performed within 3 days after the biopsy was received. Suspensions were prepared from skin biopsies as previously described [7, 8]. Genomic DNA was extracted from 2-mL suspensions using the freeze-boiling technique [9] modified as follows: five cycles of heat-cold shocks (1 min at 100°C and 1 min at –196°C in liquid nitrogen) were followed by 2 min of sonication (Bransonic Model 1200, Branson).

The diagnosis of leprosy was confirmed by smear positivity for acid fast bacilli (AFB), which were counted to establish an AFB count [10], and/or the presence of *M*. *leprae* DNA, detected by RLEP PCR, as previously published [4, 11]. In 78% of the biopsies *M*. *leprae* was detected with both methods.

The RLEP PCR was slightly modified as follows: primers (0.4 μM each) were added to beads containing dNTPS and Taq DNA polymerase (Illustra puRe Taq Ready-To-Go PCR beads, GE Healthcare). DNA in the reaction mixture was denatured for 10 minutes at 94°C and amplified during 40 cycles of 1 min at 94°C, 1 min at 50°C and 1 min at 72°C with a final extension at 72°C for 10 min. DNA samples were stored at -80°C until further analysis. The samples were anonymized and used with Institutional Review Board approval for diagnosis of specimens received at Assistance Publique–Hôpitaux de Paris, Biology laboratories of Pitié-Salpêtrière Hospital.

### SNP genotyping

DNA samples used for SNP genotyping were those used for RLEP PCR, except in the case of 15 patients where DNA was newly extracted since no amplification was obtained with thawed DNA previously used for RLEP PCR. The SNP types at positions 14 676, 1 642 875 and 2 935 685 (reference TN strain numbering system) were assessed using the original method of Monot *et al*. [6] and, when unsuccessful, using a modified protocol and primers in order to increase the efficiency of the original method [6]. Briefly, the following parameters were sequentially modified by: (i) decreasing the annealing temperature from 55°C to 48°C and the number of cycles from 45 to 30; (ii) designing new primers for nested PCR based on a combination of the original [6] and the new primers (Table 1).

**Table 1:** PCR primers used for nested PCR.

**SNP: Genome location**	Primers (5’-3’)*		Amplicon size (bp)	Annealing temperature (°C)
**14 676**	**Outer**	F_1_: CAGGTCTTGTGCGGATAA	337	51
		R_1_: AGGACACCTTCGACATGG		
	**Inner**	F_2_: AATGGAATGCTGGTGAGAGC [6]	194	56
		R_2_: CAATGCATGCTAGCCTTAATGA [6]		
**1 642 875**	**Outer**	F_3_: TGCTAGTTTAACCGAGTACTGCTA [6]	287	47,5
		R_3_: GTACAACTCATAAGCACG		
	**Inner**	F_4_: TAACTGGGCGCAGAGGAA	162	56
		R_4_: GTAGTAGTCTTCCAAGTTGTGGTG [6]		
**2 935 685**	**Outer**	F_5_: ATCTGGTCCGGGTAGGAATC [6]	268	51
		R_5_: CGTGCTGACTGTCATGAT		
	**Inner**	F_6_: TACGGTGGTGTCGGTCTC	123	56
		R_6_: ACCGGTGAGCGCACTAAG [6]		

Primers’ genome location: F1-14 492; R1-14 828; F2-14 528; R2-14 721; F3-1 642 737; R3-1 643 023; F4-1 642 764; R4-1 642 925; F5-2 935 537; R5-2 935 804; F6-2 935 594; R6-2 935 716

Crude extract (5 μL) was added to 0.5 μL of Taq DNA polymerase (1 U/μL) (bioXact long DNA polymerase, Bioline) in 2.5 μL of Taq buffer containing 5 μL of dNTP mixture (2.5 mM) (Sigma-Aldrich), 2.5 μL of 50 mM MgCl2 and 5 μL of each primer (4 μM). This reaction mixture was denatured at 94°C for 10 minutes followed by amplification consisting in 45 cycles of 1 min at 94°C for the first PCR and 30 cycles for nested PCR, 1 min at various annealing temperatures (Table 1), 2 min at 68°C and a final extension at 68°C for 10 min. In case of nested PCR, products of first PCR was applied to second PCR without dilution. After DNA amplification, unincorporated nucleotides and primers were removed by filtration through the membrane of a 96-well MultiScreen Filter Plate (Merck Millipore Ltd.) placed onto a MultiScreen _HTS_ Vacuum Manifold connected to a vacuum pump. Sequencing reactions were carried out in a final volume of 10 μL, with 0.8 μL of Big Dye Sequencing RR-100 (Big Dye Terminator Cycle Sequencing Ready reaction kit (Applied Biosystems)), 2 μL of BigDye sequencing buffer 5x, 0.6 μL of forward or reverse primers 4 μM and 2 μL of purified PCR product. The cycle sequencing was carried out on a thermal cycler using the following conditions: 1 min of initial denaturation at 96°C, 25 cycles of the following program segment: 10 sec at 96°C, 5 sec at 50°C, 4 min at 60°C. Sequencing products were purified with a Sephadex G-50 (GE Healthcare) gel filtration system. Sequences were compiled and analyzed using BioEdit software, as previously described [12].

## Results

### Efficiency of SNP genotyping protocols

Four SNP types were identified in this study. Applying the original method of Monot *et al*. [6] allowed their identification in 22/71 isolates, decreasing the annealing temperature and the number of cycles resulted in the characterization of an additional 22 isolates and nested PCR allowed the characterization of a further 23 isolates. Overall, 67/71 isolates (94%) were successfully genotyped. For the 4 isolates unsuccessfully genotyped, even newly extracted DNA did not allow SNP genotyping.

### Geographic origins of the 67 patients for whom the *M*. *leprae* strain was genotyped

Thirty-five of the 67 patients (52%) for whom the *M*. *leprae* strain was successfully genotyped were born in French overseas territories: five in Mayotte, 21 in New Caledonia, four in French Polynesia, four in French West Indies and one in La Réunion. Twenty-eight patients (42%) were immigrants living in France: 16 from Comoros, two from Guinea, and one each from Mali, Ivory Coast, Benin, the Democratic Republic of the Congo (DRC), the Republic of the Congo (RC), Madagascar, Haiti, Brazil, India and Sri Lanka. The four remaining patients (6%) were born in metropolitan France; two of them are living or have lived for a long time in RC or DRC, one has been travelling frequently in India and one was a humanitarian worker in many countries, including leprosy endemic areas.

### SNP genotype analysis

**Fig 1:**
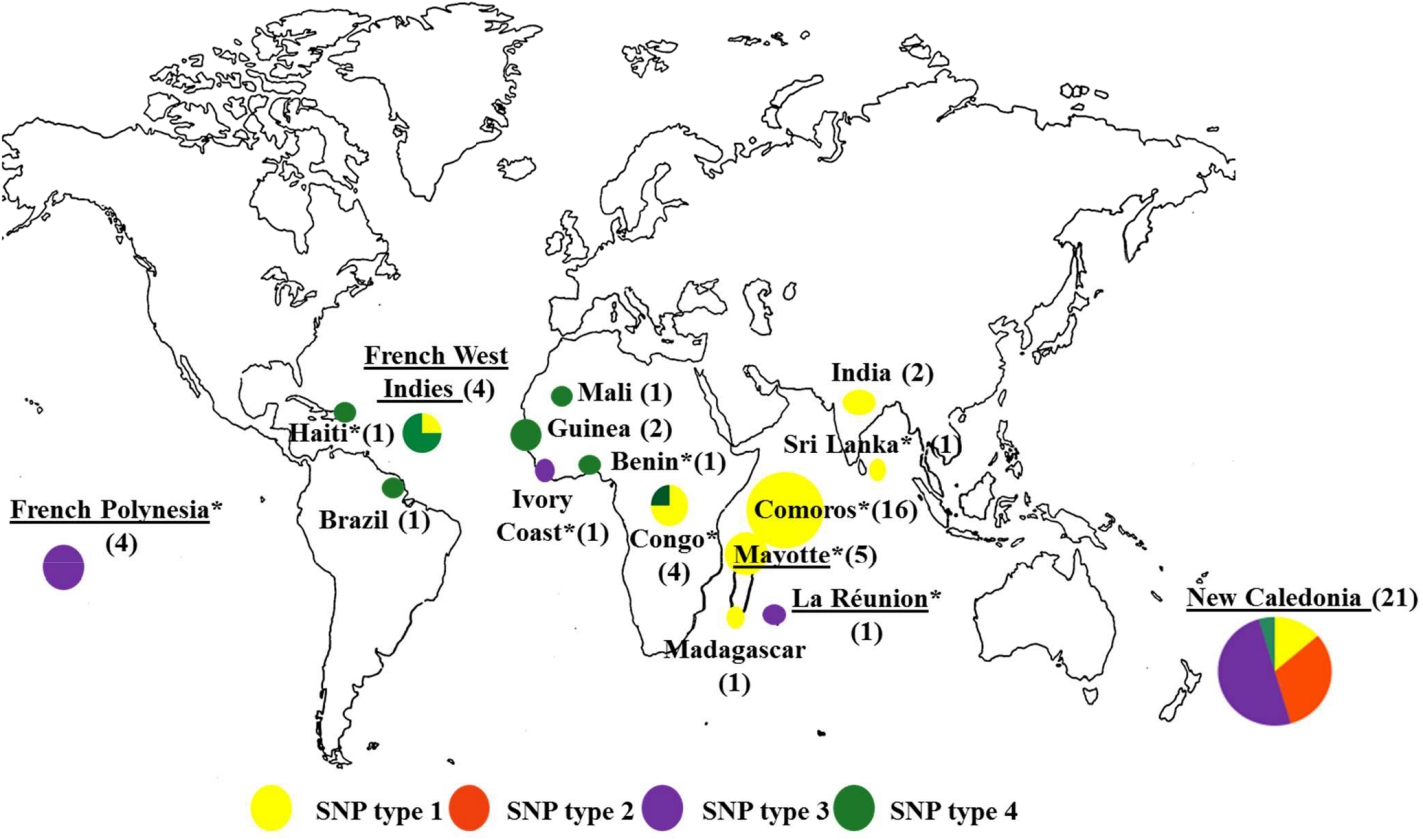
Leprosy cases diagnosed in France (2009–2013): correlation between SNP genotype and geographical origin of the patient. (Total = 66 cases, for 1 one of the 67 cases included in our study the country where leprosy was acquired in unknown). *indicates countries for which no previous SNP data were available. Underlined countries indicate the French overseas territories. For Congo: 1 patient was born in DRC, 1 patient was born in RC, 1 patient was born in France but has lived in DRC and 1 patient was born in France but has lived in Congo without further information.

SNP type 1 was predominant (33 cases), followed by SNP type 3 (17 cases), SNP type 4 (11 cases) and SNP type 2 (6 cases) (Fig 1).

Among the cases in which several biopsies were examined (i.e. two biopsies from six patients and three biopsies from two patients), the SNP types were concordant in each individual case.

The majority of the 35 patients from French overseas territories were from New Caledonia (n = 21) and were infected with isolates belonging to SNP type 1 (n = 3), SNP type 2 (n = 6), SNP type 3 (n = 11) and SNP type 4 (n = 1). The other patients of this group were from French Polynesia (n = 4; SNP type 3), Mayotte (n = 5; SNP type 1), the French West Indies (n = 4) including Guadeloupe (n = 1; SNP type 1) and Martinique (n = 3; SNP type 4) or La Réunion (n = 1; SNP type 3).

Among the 28 immigrant patients, those infected with SNP type 1 were born in RC (n = 1), Comoros (n = 16), India (n = 1), Sri Lanka (n = 1) and Madagascar (n = 1). Patients with SNP type 3 were from Ivory Coast (n = 1) and those with SNP type 4 were born in Mali (n = 1), Guinea (n = 2), Benin (n = 1), DRC (n = 1), Brazil (n = 1) or Haiti (n = 1). The isolates from the four remaining patients who were born in metropolitan France were of SNP type 1.

## Discussion

Between 20 and 30 leprosy cases are still diagnosed each year at the French NRC. We investigated the correlation between the geographic origin of the patients and the SNP type of the *M*. *leprae* isolates collected over a period of five years.

In general, the choice of the markers used for genotyping has been dependent on the timescale of the events of interest, with rapidly evolving markers (VNTR markers) for contact tracing over relatively short periods of time, and slower evolving markers (SNPs) for tracing dissemination of strains over hundreds to thousands of years [13]. The latter marker type was appropriate for our goal.

By using several protocols, including nested PCR, the genotype of 94% of the isolates analyzed in the study has been determined. Amplifying DNA from clinical specimens for retrospective studies after freeze-thaw cycles can be difficult. Information on the yield (success rate) is usually lacking in publications reporting on *M*. *leprae* genotyping and where it was specified, it was of the order of 75% [14] or 94% [15]. Although using three different methods, i.e. (i) the original method of Monot *et al*. [6], (ii) modified PCR conditions and (iii) nested PCR, we could not amplify DNA from four biopsies that were either AFB-negative or with low AFB counts (< 8 ×10^4^ AFB/ml). In contrast, the mean AFB count was 7×10^6^/ml in the skin biopsy suspension containing isolates whose SNP type was successfully determined, in agreement with a study reporting that DNA amplification efficiency increases with increasing AFB counts [7].

Most leprosy patients diagnosed in France were born outside metropolitan France, either in another country (n = 28) or in French overseas territories (n = 35), whereas only four patients were born in metropolitan France. France is a country of 65 million inhabitants living either in metropolitan France or in French overseas territories (2 million inhabitants), scattered over six continents. In the latter territories, leprosy is not eradicated and in 2011 the prevalence of leprosy (per 10 000 inhabitants) was 3.72 in Mayotte, 0.097 in La Réunion, 0.155 in Guadeloupe, 0.025 in Martinique, 0.7 in French Polynesia and 0.47 in New Caledonia [16].

Similar data have been reported in other Western European countries. In England, a review of 80 leprosy cases in Liverpool and Birmingham diagnosed between 1946 and 2003 showed that all patients except one acquired leprosy on the Indian subcontinent, in Africa or in South America. The only case of leprosy transmission within the UK concerned a 9-year-old child who acquired the disease in the 1940s from his father who had been infected in Brazil [17]. In Germany two cases were reported in 2006 of patients born in Pakistan or Sri Lanka [18], whereas in Italy 59 cases were reported between 2003 and 2009, all of patients who had immigrated from various countries [19]. In Spain, seven cases were described between 2004 and 2009, six of immigrants and one of a Spanish patient who had worked for 25 years as a missionary in Venezuela [20]. WHO did not report European leprosy cases in its last report in 2013 [2].

The main results of the present study were (i) the demonstration that the SNP genotype of the isolates from leprosy patients born in metropolitan France correlated with the type(s) encountered in the countries these patients had visited or in which they had lived and where leprosy is not eradicated, (ii) the genotype determination of isolates originating from regions for which no data were available so far and (iii) the augmentation of data for regions covered by already published studies.

For the patients who were born outside metropolitan France, i.e. in French overseas territories or other countries for which published records of *M*. *leprae* SNP types exist, the types correlated well with those encountered in the countries of birth of 31 patients (Fig 1). Published data in conjunction with the data of the present study are presented in Table 2 to provide a comprehensive overview of *M*. *leprae* SNP types classified according to geographical regions. There was a single incoherence, concerning the SNP type 3 isolate from a patient born in Ivory Coast, a type previously observed in North Africa and the Americas but so far not in West Africa where SNP type 4 is prominent. The latter type was the only one found in six patients born in Ivory Coast and 31 patients born in Mali (Table 2). We found a SNP type 4 for the first time in a patient born in New Caledonia where types 1, 2 and 3 have been observed previously (Fig 1, Table 2), likely reflecting diversity of populations and origins of migration flow, especially among the French overseas territories including the French West Indies where SNP type 4 is prominent.

**Table 2:** Review of *M*. *leprae* SNP types previously described in different countries.

WHO Region	Country	SNP type 1		SNP type 2		SNP type 3		SNP type 4	
			Total		Total		Total		Total
**African**	Mali							31[6]+1**§**	32
	Sénégal							2[6]	2
	Guinea							1[6]+2**§**	3
	Ivory Coast*					1**§**	1	6[6]	6
	Benin*							1**§**	1
	Congo*	3**§**	3					1**§**	1
	Ethiopia			2[6]	2				
	Malawi	4[6]	4	6[6]	6				
	Comoros *	16**§**	16						
	Mayotte *	5**§**	5						
	Madagascar	6[6]+1**§**	7						
	La Reunion *					1**§**	1		
**American**	United States					3[6]	3		
	Mexico	6[21]	6	13[21]	13	45[21]+1[6]	46		
	Venezuela					5[6]	5		
	Brazil	4[22]	4			37[22]+12[6]	49	46[22]+2[6]+1**§**	49
	Haiti*							1**§**	1
	French West Indies *	4[6] +1**§**	5			2[6]	2	14[6]+3**§**	17
	Bolivia, Brazil, Uruguay, Venezuela	16[23]	16			172[23]	172	45[23]	45
**South East Asia**	Korea	3[6]+15[24]	18	4[24]	4	2[6]+17[24]+7[25]	26		
	India	166[26]+27[6]+11[14]+45[15]+1**§**	250	14[26]+5[6]	19				
	Sri Lanka*	1**§**	1						
	Indonesia	12[24]	12	7[24]+1[25]	8	16[24]	16		
	Myanmar	26[24]	26	1[24]	1	2[24]	2		
	Thailand	1[6]+2[25]+68[27]	71			17[27]	17		
**Eastern Mediterranean **	Morocco					2[6]	2		
**Western Pacific**	China	18[28]	18	1[28]	1	82[28]	82		
	Japan	1[25] +4[24]	5	2[24]	2	16[25]+47[24]	63	2[24]	2
	Philippines	19[6]+ 175[29]	194	6[25]	6	2[6]+ 32[29]	34		
	New Caledonia	3[6]+3**§**	6	1[6] +6**§**	7	3[6] +11**§**	14	1**§**	1
	French Polynesia *					4**§**	4		

* Indicates countries for which no previous SNP data were available

§ countries/territories covered in this study

French overseas territories are underlined

Concerning Congo: one patient was born in DRC, one patient was born in RC, one patient was born in France but has lived in DRC and one patient was born in France but has lived in Congo (without further information).

Furthermore, we determined SNP types of isolates from patients born in countries or regions not covered by previous studies (n = 31), i.e. Comoros, Haiti, Sri Lanka, Benin, Congo (RC and DRC), La Réunion, Mayotte and French Polynesia. In a patient born in Haiti we found SNP type 4 which is not surprising since this type is prominent in several countries of South America (i.e. Brazil, Bolivia, Uruguay, Venezuela and Mexico (Table 2)). In a patient born in Sri Lanka, we found SNP type 1, the major type found in India, a geographically close country (Table 2). In one patient born in Congo (DRC) and one patient born in Benin, we found SNP type 4, in agreement with previous data describing this type in West African countries but also one case of SNP type 1 in Congo (RC), a type found so far in Eastern Africa. However, it should be noted that the number of SNP typed *M*. *leprae* isolates from Africa, with the exception of Mali, is rather small (Table 2). In Mayotte and Comoros we found only SNP type 1 which is not surprising regarding the geographic localization of these islands between Madagascar and India where SNP type 1 is prominent.

We found only SNP type 3 in La Réunion (n = 1) and French Polynesia (n = 4) (Fig 1). For Polynesia this is not surprising given the history of its settlement and its location in the Western Pacific region where SNP type 3 is common. As for La Réunion, the result can be explained by the immigration at the end of the 19^th^ century of a large group of settlers, locally called “Sinwa”, from Southern China where SNP type 3 is prevalent (Table 2).

The regions in which three of the four patients born in metropolitan France were infected are reasonably obvious: two lived in Congo, therefore the SNP type 1 found in both cases and previously described in Congo (RC and DRC; Fig 1) supports the acquisition of leprosy in that region. One patient also infected with an SNP type 1 strain lived in India, another country where this type is frequently described (Table 2). As to where the last patient was infected, a humanitarian worker active in many countries, including leprosy endemic areas, we do not have enough information to support speculation.

The phylogenic analysis of *M*. *leprae* strains isolated in France showed a good correlation between *M*. *leprae* genotypes and the geographical origin of the patients. These results are in agreement with the view that French leprosy cases are acquired in regions where leprosy is still endemic (countries other than France) or present (French overseas territories) but not in metropolitan France where leprosy has been eradicated.

In conclusion, the SNP types of all *M*. *leprae* isolates from leprosy cases diagnosed in metropolitan France from 2009 to 2013 were consistent with what is known about their distribution in different regions of the world. The phylogenetic analysis also showed agreement between *M*. *leprae* genotype and geographical origin of the patients. The analysis further expanded our knowledge regarding the strain types from French overseas territories and their connection with migration flow. Previously published data combined with those reported here indicate that, in a globally stable situation, major changes in the prevalence of SNP types occur in regions where migration flow is greatest.
